# The world trade network: country centrality and the COVID-19 pandemic

**DOI:** 10.1007/s41109-022-00452-4

**Published:** 2022-03-21

**Authors:** Roberto Antonietti, Paolo Falbo, Fulvio Fontini, Rosanna Grassi, Giorgio Rizzini

**Affiliations:** 1 Department of Economics and Management, University of Padova, Via del Santo 33, 35123 Padova, Italy; 2 Department of Economics and Management, University of Brescia, Contrada S. Chiara 50, 25122 Brescia, Italy; 3 Department of Statistics and Quantitative Methods, University of Milano - Bicocca, Via Bicocca degli Arcimboldi, 8, 20126 Milan, Italy

**Keywords:** World trade network, Centrality measures, Community detection, COVID-19, 91B60, 05C82, 62P20

## Abstract

International trade is based on a set of complex relationships between different countries that can be modelled as an extremely dense network of interconnected agents. On the one hand, this network might favour the economic growth of countries, but on the other, it can also favour the diffusion of diseases, such as COVID-19. In this paper, we study whether, and to what extent, the topology of the trade network can explain the rate of COVID-19 diffusion and mortality across countries. We compute the countries’ centrality measures and we apply the community detection methodology based on communicability distance. We then use these measures as focal regressors in a negative binomial regression framework. In doing so, we also compare the effects of different measures of centrality. Our results show that the numbers of infections and fatalities are larger in countries with a higher centrality in the global trade network.

## Introduction

At the beginning of 2020, the COVID-19 disease rapidly spread from the local Chinese region of Hubei, soon becoming a global health emergency. Since it originated in a highly populated region that is strategic for several industrial sectors, the effects of lockdown restrictions led to a freezing of business investments and a reduction in Chinese households consumption, which had a significant impact on Chinese trades. The spread of COVID-19 has rapidly and severely affected every economy in the world.

Understanding the factors that triggered the COVID-19 outbreak is still an subject of debate. The spread of a pandemic is a complex matter, affected by several interacting elements. On top of physical elements, such as, for instance, temperature, humidity, and air pollution, socio-economic factors seem to have played a major role in driving the COVID-19 outbreak (Antonietti et al. 2021b). The reasons and opportunities that compel people to travel around the world to spend time meeting other people have sustained the chances for the virus to spread. Different kinds of business, social and/or family reasons determine much more effectively the chances for people to enter into physical contact with each other at both the global and local scales. An estimation of the impact of socio-economic factors on the spread of the COVID-19 pandemic is therefore of clear importance.

Some of research contributions related to the COVID-19 pandemic are concerned with models that aim to describe and understand the dynamics of the pandemic, that is, how it has spread over time as a consequence of a series of variables considered as exogenous. Epidemic models clearly belong to this class. A different class of models looks to static or preliminary conditions that can be related to the huge differences in the diffusion of the consequences of the pandemic. Our contribution falls into this last framework. More precisely, we aim at identifying the significant properties of the set of world trades that can explain the initial differences across countries with respect to the infection and death rates. The two points of view positively interact, since all system dynamic models depend on the initial conditions to explain future evolution. This is particularly true in the case of the COVID-19 pandemic. Indeed, starting from the World Health Organization’s (WHO) declaration, the evolution of COVID-19 in each country followed independent and autonomous paths, driven mostly by more or less severe lockdown policies, which dramatically reduced the usual volume of global trade and the mobility of people. So, if any link exists between world economic exchanges and the COVID-19 pandemic, it can first be traced out during its early stages, since in the following periods the adoption of diversified containment policies by national governments can make the role of international trade less clear.

In this paper, we stress the importance of countries’ central positions in the global trade scenario. From a topological point of view, the economic transactions between countries are characterized by an intricate weave of relations, and complex network theory offers an effective representation of this situation. Both connections between countries and bilateral trade flows can be modelled as a dense network of interconnected agents. However, a major difficulty arises in the search for such interconnections. More precisely, since the trade network is naturally dense and almost complete, the study of the classical global network indicators applied to the whole network is not informative enough. This demands an accurate choice of more effective network tools. In order to make this choice, we first assess countries’ centrality, identifying a satisfactory representation of the international trade landscape. Focusing on 2019 and 2020, such analyses allow us to detect whether any change in the international trade network has occurred and relate it to the emergence of COVID-19, focusing on those measures that are meaningful in capturing possible modifications. We then assess whether these centrality measures have an explanatory power with respect to the wide differences in the rates of infection and mortality that have been observed worldwide, once a series of other confounding factors have been controlled for. We stress that our aim is not to identify possible network measures that can be used as proxies of the spread of COVID-19, but rather to specify the role that the complex structure of the world trade network (WTN) has played during the beginning of the pandemic. Hence, we detect the presence of trade communities not only via their direct connections, as measured by the total volume of trade directly exchanged between two countries, but also via indirect connections. Indeed, we argue that it is crucial to consider deep interconnections between nodes to capture strategic commercial links, which can survive beyond a global shock. To this end, we apply the recent methodology proposed by Bartesaghi et al. (2020), focusing on the Estrada communicability distance (Estrada and Hatano 2009). As a result, the analysis of communities performed on 2019 and 2020 shows that the trade network is a resilient structure, adapting itself to a global shock such as a pandemic. We provide strong empirical evidence that, on the contrary, network centrality measures can explain the early diffusion and mortality rates of COVID-19. We show, in particular, that a higher country centrality in the WTN corresponds to a higher risk of infection and death. In addition, the community clustering coefficient, which synthesises both the community structure and countries’ centrality, can explain the high number of deaths and infections better than the classical clustering coefficient.

The paper is organised as follows: in “Related literature” section, we review the relevant literature. “Methodology and network indicators” section describes the methodology and the network indicators, as well as the econometric model used to perform the analysis. In “Data, samples and variables” section, we describe the WTN and socio-economic data used and how we constructed the WTN. In “Results” section, we report and discuss the results of the network analysis and the econometric model. Conclusions follow in “Conclusions” section. “Appendix A” reports the list of countries used to develop the analysis. “Appendix B” shows the WTN visualisations for 2019 and 2020. “Appendix C” shows the community detection obtained through the modularity optimisation (Louvain method).

## Related literature

Some works in the literature relate the level of mobility of people (both at the global and local levels) to the COVID-19 pandemic (Antonietti et al. 2021a; Fernández-Villaverde and Jones 2020). More precisely, countries with higher levels of inward international mobility have higher probabilities of anticipating the time of the first contagions and having a higher number of infected people freely circulating during the pre-symptom period. Russo et al. (2020) point to January 18th as day zero of the COVID-19 outbreak in Lombardy (Italy), which has been one of the most severely hit regions worldwide. Parodi and Aloisi (2020) suspect that the abnormal number of cases of bilateral pneumonia that occurred in Lombardy already in December 2019 could be attributed to COVID-19. A factor that increases the probability of early contagion in a region, or a country, is certainly the movement of the citizens outside and inside its borders. In their cross-sectional analysis based on Spanish regions, Paez et al. (2020) observe that local public mass transportation systems, more than international airport facilities, appear to be linked to a higher severity of contagion rates. International and local transportation seem to act differently. The former increases the chances of early contagion events, while the latter acts as a second-order contagion enhancer.

International trade data can be used as a comprehensive indicator accounting for population density, economic dynamism and human mobility. In this regard, Bontempi and Coccia (2021) investigate the relations between the total imports and exports of 107 provinces in Italy and COVID-19 transmission dynamics. Extending previous work, Bontempi et al. (2021) focus on regional data from France, Italy and Spain and confirm the relevance of trade in the analysis of the COVID-19 pandemic, finding a strong positive correlation between the international trade volume of each region and the percentage of patients who recovered in intensive care units. From a network perspective, the impact of topology and metric properties on the stability and resilience of an economic or financial system has been widely studied in the literature (see e.g. Kali and Reyes 2007; Piccardi and Tajoli 2018).

On the one hand, community detection is a useful tool to see how an external shock modifies the topological structures of complex systems (Fortunato and Hric 2016). On the other hand, a suitable metric can highlight the role of non-local interactions between nodes. In this regard, Estrada and Hatano (2008, 2009) introduce the concept of communicability, presenting a metric between nodes that takes into consideration long-range interactions between them.

An area in which these concepts allow us to gain a deep insight into the hidden structures of the network is properly the WTN (see Bartesaghi et al. 2020).

The topology of the WTN has been extensively analysed over time. The behaviour of international trade flows, the impact of globalisation on international exchanges, the presence of a core-periphery structure and the evolution of community centres of trade are just some of the issues addressed by recent developments (see Serrano et al. 2007; Tzekina et al. 2008; Fagiolo et al. 2010; De Benedictis and Tajoli 2011; Blöchl et al. 2011; Grassi et al. 2021; Ercsey-Ravasz et al. 2012). Recently, some works have correlated commercial trade with COVID-19 diffusion from a network point of view (Antonietti et al. 2020; Reissl et al. 2021; Kiyota 2022; Fagiolo 2020; Gruszczynski 2020).

Such results further motivate the analysis of the link between the COVID-19 pandemic and the trade networks between countries.

## Methodology and network indicators

In this section, we briefly introduce the definitions of the centrality measures used later for the econometric analysis. We then describe the methodology that we apply in the paper. In particular, we use the community detection method based on the Estrada communicability distance, recently proposed in Bartesaghi et al. (2020). We present the main steps of the methodology and we refer the reader to the cited reference for a detailed description.

The application of centrality measures, as well as the study of the network topology of the WTN, are useful in explaining the initial diffusion of the pandemic, as explained in “Related literature” section. The origins of COVID-19 are still largely uncertain, as well as the very early stages of its spread outside China. However, it can be supposed that, during this first period, the chances of “importing” the SARS-COV-2 virus were not the same for all countries. On the contrary, the established trade routes for the circulation of commodities and the mobility of people have probably driven the direct or the indirect import of the SARS-COV-2 virus inside national borders, determining significant initial differences in the early contagion rates between entire clusters of countries.

These reasons motivate the use of centrality measures and of the Estrada communicability distance in explaining the first wave of the contagion. On the one hand, the Estrada communicability distance allows us to highlight the strategic commercial links, which can survive beyond a global shock. On the other hand, suitable centrality measures quantify specific factors, such as the number and volume of trades, the power of a country in commercial framework and triadic relations between countries, which are non-negligible in studying the initial diffusion of the pandemic.

From now on, we consider a simple weighted undirected network $G=(V,E),$ where *V* is the nodes set with $|V| = n$ and *E* is the set of links. The unweighted and weighted adjacency relations are represented by matrices ${\mathbf {A}}$ and ${\mathbf {W}}$, respectively.

### Centrality measures

Many centrality measures have been proposed in the literature. Among them, some measures highlight various characteristics of the WTN.

The first measure we use in the analysis is the most intuitive one, i.e. the degree centrality. This measure counts the number of links incident on a vertex. For weighted networks, the corresponding measure is the strength centrality. In the WTN, these measures quantify how much a country directly trades, in terms of number and volume of trades.

The eigenvector centrality [see Bonacich (1972)] is formally represented by the *i*-th component of the principal eigenvector of the adjacency matrix. Since it quantifies the connections of a vertex with its neighbours that are themselves central, it can be interpreted as a measure of the power of a country in the trading scenario. The extension to the weighted case is immediate, as the weighted adjacency matrix ${\mathbf {W}}$ preserves all characteristics of ${\mathbf {A}}$.

The betweenness centrality of a node is the fraction of the shortest paths between pairs of nodes passing through it. With reference to the WTN, with trade many other elements are transferred between countries. In this perspective, this measure quantifies the influence that a country has in spreading information within the network.

We also consider the local clustering coefficient, which measures the tendency to which nodes in a network tend to cluster together. Since the WTN is represented by an indirect and weighted network (see “Data, samples and variables” section for a detailed description of the network), we focus on the local weighted coefficient proposed by Onnela et al. (2005):

1 $$\begin{aligned} C_i(\tilde {{\mathbf{W}}}) = \frac{\sum _{j} \sum _{j\ne k} {\tilde{w}}_{ij}^{1/3} {\tilde{w}}_{jk}^{1/3} {\tilde{w}}_{ki}^{1/3}}{d_i(d_i -1)} \end{aligned}$$

where $d_i$ is the degree of node *i* and $\tilde {{\mathbf{W}}}$ is the weighted adjacency matrix obtained by normalizing the entries $w_{ij}$ of ${\mathbf {W}}$ as ${\tilde{w}}_{ij} = \frac{w_{ij}}{\max (w_{ij})}$ $\forall i,j$. Notice that $C_i(\tilde {{\mathbf{W}}})=C_i$ represents the geometric mean of the links weights incident to the node *i*, divided by the number of potential triangles $d_i(d_i-1)$ centred on it. The main idea is to replace the total number of triangles in which a node *i* belongs with the “intensity” of the triangle, defined here as the geometric mean of its weights. Since it is a measure of how many nodes are locally clustered, the clustering coefficient is extremely interesting to investigate in the context of international trade. Indeed, trade relationships induce a dependency between countries, as two nodes that are both trading partners of a node are likely to trade themselves. From this perspective, it is interesting to investigate how countries are reciprocally dependent, that is, how nodes are clustered together.

### Community detection based on communicability distance

The main idea is to detect communities by optimising a quality function that exploits the additional information contained in a metric structure based on the Estrada communicability. At first, we recall the definition of the Estrada communicability (simply, communicability) between two nodes *i* and *j* (see Estrada and Hatano (2008)):

2 $$\begin{aligned} G_{ij}=\sum _{k=0}^{+\infty }\frac{1}{k!}[\mathbf{A }^k]_{ij}=\left[ e^{\mathbf{A }} \right] _{ij}. \end{aligned}$$

As the *ij*-entry of the *k*-power of $\mathbf{A }$ provides the number of walks of length *k* starting at *i* and ending at *j*, $G_{ij}$ accounts for all channels of communication between two nodes, giving more weight to the shortest routes connecting them. The elements $G_{ii}$, $i=1,\ldots ,n$ are known in the literature as subgraph centrality (Estrada and Rodriguez-Velazquez 2005). The communicability matrix is, then, the exponential of the matrix ${\mathbf {A}}$, simply denoted by $\mathbf{G }$.

In the case of a weighted network, the weighted communicability function is defined as

3 $$\begin{aligned} G_{ij}=\sum _{k=0}^{+\infty }\frac{1}{k!}[(\mathbf{S }^{-{\frac{1}{2}}}\mathbf{W }\mathbf{S }^{-{\frac{1}{2}}})^k]_{i j}=\left[ e^{(\mathbf{S }^{-{\frac{1}{2}}}\mathbf{W }\mathbf{S }^{-{\frac{1}{2}}})} \right] _{ij} \end{aligned}$$

where $\mathbf{S }$ is the diagonal matrix whose diagonal entries are the strengths of the nodes. Following Crofts and Higham (2009), the matrix $\mathbf{W }$ in Formula (3) has been normalized to avoid the excessive influence of links with higher weights in the network.

Using the communicability, a meaningful distance metric $\xi _{ij}$ can be constructed, as defined in (Estrada 2012):

4 $$\begin{aligned} \xi _{i j}=G_{ii}-2G_{ij}+G_{jj}. \end{aligned}$$

By definition, communicability measures the amount of information transmitted from node *i* to *j*. On the other hand, $G_{ii}$ measures the importance of a node according to its participation in all closed walks to which it belongs. Hence, in terms of information diffusion, $G_{ii}$ is the amount of information that, after flowing along closed walks, returns to node *i*.

Thus, the quantity $\xi _{ij}$ accounts for the difference in the amount of information that returns to nodes *i* and *j* and the amount of information exchanged between them. The greater is $G_{ij}$, the larger is the information exchanged and the nearer are the nodes; the greater are $G_{ii}$ or $G_{jj}$, the larger is the information that comes back to the nodes and the farther are the nodes. Since $\xi _{i j}$ is a metric, then $G_{ii}+G_{jj}\ge 2G_{ij}$, i.e. no matter what the structure of the network is, the amount of information absorbed by a pair of nodes is always larger than or equal to the amount of information transmitted between them.

This metric is meaningful if we apply it to the WTN. Indeed, network flows along links measure how well two countries communicate in terms of commercial exchanges. For instance, the link between two nodes may be identified with the total trade or money flow between two countries.

We assume that two nodes are considered members of the same community if their mutual distance $\xi _{ij}$ is lower than a threshold $\xi _0 \in [\xi _{min},\xi _{max}]$. In particular, we construct a new community graph with adjacency matrix $\mathbf{M }=[m_{ij}]$ given by:

5 $$\begin{aligned} m_{ij}= \left\{ \begin{array}{ll} 1 &{} \ \mathrm{if}\ \xi _{ij}\le \xi _0 \\ 0 &{} \ \mathrm{otherwise} \\ \end{array} \right. \end{aligned}$$

In this way, clustered groups of nodes that ’strongly communicate’ emerge, varying the threshold $\xi _0$.

As well explained in Bartesaghi et al. (2020), $\xi _0$ is not arbitrarily chosen but is obtained by solving the following optimisation problem:

$$\begin{aligned} \xi _0 \in \arg \max Q. \end{aligned}$$

The objective function *Q* is

6 $$\begin{aligned} Q = \sum _{i,j} \gamma _{ij}x_{ij}, \end{aligned}$$

where $x_{ij}$ is a binary variable equal to 1 if nodes *i* and *j* belong to the same community and 0 otherwise. $\gamma _{ij}$ is a function measuring the cohesion between nodes *i* and *j*. Originally proposed in Chang et al. (2016), it is defined in Bartesaghi et al. (2020) as follows:

7 $$\begin{aligned} \gamma _{ij} = ({\bar{\xi }}_j - {\bar{\xi }}) - (\xi _{ij}- {\bar{\xi }}_i), \end{aligned}$$

where ${\bar{\xi }}_{j}$ is the average distance between node *j* and nodes other than *j* and ${\bar{\xi }}$ is the average distance over the whole network.

Since two nodes are cohesive (and incohesive, respectively) if $\gamma _{ij}\ge 0$ $(\gamma _{ij}\le 0)$, in terms of distance, they are cohesive if they are close to each other and, on average, they are both far away from the other nodes.

From this perspective, $\gamma _{ij}$ can be seen as the ’gain’ if positive or the ’cost’ if negative of grouping two nodes *i* and *j* in the same community. The applied methodology will allow us to discover communities in the WTN based on all the possible channels of interactions and exchanges between countries.

### Econometric model

#### Baseline model

In what follows, we want to assess the role of the WTN in the evolution of the pandemic in the five weeks between March 11th and April 21st, 2020. At the same time, we want to control for additional socio-economic factors that can have an impact on the diffusion of the pandemic. To avoid the possibility that, in turn, these factors might be affected by COVID-19 diffusion, we include them as referring to 2019.

The baseline model that we adopt to test for the role that network centrality has played in explaining the number of infections (INF) and deaths (DEATH) in the first wave of the COVID-19 outbreak (i.e. between March 11st, 2020 and April 21st, 2020) is the following:

8 $$\begin{aligned} Y_{it}=\beta _0 + \beta _{1,i} TNC_i+{\mathbf {Z}}'_i{\varvec{\beta }}_Z+ \gamma _t + \epsilon _{it} \end{aligned}$$

where $$Y_{it}$$ is either the number of COVID-19 infections (INF) or the number of deaths (DEATH) in country *i* and week *t*. The variable $$TNC_{i}$$ stands for trade network centrality and represents a given centrality measure^1^ (respectively: degree, strength, weighted eigenvector and weighted clustering coefficient) measured in 2019; $${\mathbf {Z}}$$ is a vector of additional regressors that can explain the number of infections and fatalities due to COVID-19, namely GDP per capita (GDPPC, at constant 2010 US$), total resident population (POP), the share of elderly population (POP65+), the number of hospital beds per 1,000 inhabitants (HBEDS) and the average temperature in February and March (TEMP) in degrees Celsius, all measured in 2019. The term $$\gamma _t$$ is a series of five week-specific dummies that capture the trend in the dynamics of COVID-19 infections and fatalities for all our countries,^2^ while $$\epsilon _{it}$$ is the stochastic error component with zero mean and finite variance $$\sigma ^2_{\epsilon }$$. To control for the unobserved arbitrary within-group correlation of our observations, we cluster the standard errors at the country level.

Since $Y_{it}$ is a count variable, and our regressors are time-invariant because they are all measured in 2019, we estimate Eq. (8) using a pooled negative binomial regression model. As is common for count-data models, we test for the overdispersion of our data, that is, for the fact that the conditional mean can be lower than the conditional variance, typically due to the presence of unobserved factors than can affect the number of COVID-19 infections or deaths. In such a case, the main assumption for the use of the Poisson model is violated, and the negative binomial model fits the data better.

We also check for the presence of potential multicollinearity by re-estimating Eq. (8) through a linear regression model and using a variance inflation factor (VIF) statistic. 3 Multicollinearity can be considered an issue if the VIF statistic takes a value higher than the commonly accepted threshold of 5. To check which of the proposed trade network centrality measures provides the highest explanatory power in predicting $Y_{it}$, we use the Akaike information criterion (AIC) and Bayesian information criterion (BIC).

To compare the magnitude of the estimated coefficients, we standardise all the regressors by subtracting their mean and dividing by their standard deviation. For each variable, we report the incidence rate ratio (IRR), which measures the impact of a unit increase of the regressor on the risk of contagion (mortality) from COVID-19, computed as the ratio between the number of infected (deceased) individuals and the number of non-infected (surviving) individuals. In this respect, the IRR of a regressor is easier to interpret than the corresponding estimated coefficient, since the latter measures the impact of a unit increase in the regressor itself on the log of the expected number of infections or deaths. We also test for the validity of our negative binomial regression mode in two ways. First, we estimate Eq. (8) using a Poisson model, and we use the Pearson goodness of fit test, where a significant $\chi ^2$-distributed statistic would reveal that, because of overdispersion in the data, the Poisson regression model is not appropriate, and a negative binomial specification should be preferred. Second, after estimating Eq. (8), we compute the average predicted probabilities and we compare the observed number of infections and deaths with the number predicted by our negative binomial regression model.

#### Econometric model considering WTN mesoscale structure

To check whether the WTN community structure had an impact on COVID-19 diffusion during the first wave, we re-estimate Eq. (8) using an averaged local clustering coefficient of network communities detected with the methodology described in “Community detection based on communicability distance” section. We then compare the IRRs with those estimated for the local clustering coefficient (as in Eq. (1)). Specifically, for each community, we compute the average of the clustering coefficients ${\bar{C}}$ of the countries therein. Therefore, each country in community *k* has a new clustering coefficient equal to ${\bar{C}}_{k}$, defined by

9 $$\begin{aligned} {\bar{C}}_{k} = \frac{1}{n_k} \sum _{j=1}^{n_k} C_{j} \end{aligned}$$

where $n_k$ is the size of community *k* and $C_{i},$ is the local clustering coefficient of node *i* as in Eq. (1).

Coefficient ${\bar{C}}$ has two properties: on the one hand, it still reflects the country’s centrality within all its triadic relations expressed by the local clustering coefficient in Eq. (1). On the other hand, ${\bar{C}}$ takes into account the mesoscale structure of the WTN based on communicability. In other words, with this new coefficient, we capture the impact of a country’s centrality in a subset of the WTN, where nodes strongly exchange trade-related information that can be directly observable (such as merchandise trade) or indirectly observable (such as the interactions characterising the supply chain of a good).

We then re-estimate Eq. (8) using as a network centrality measure the average community local clustering ${\bar{C}}_k,$ as in Eq. (9), and we compare the newly estimated IRR with that of the local clustering coefficient of each country. We also provide a series of robustness tests in which we re-estimate Eq. (8) week by week, dropping the term $\gamma _t$ and using a series of five distinct cross-sectional negative binomial regression models for each of the two dependent variables, INF and DEATH, respectively. In this way, we can observe whether, and to what extent, the estimated IRRs vary along the first wave of the COVID-19 pandemic, and test for the stability of the IRRs for the country-specific network centrality measures, as compared with the corresponding community-level measures.

## Data, samples and variables

### Dataset description

The empirical analysis is based on two datasets. The first is used to construct the network and consists of a sample of monthly trade values during the first semesters of 2019 and 2020 for 55 countries^4^ listed in Table 11 of “Appendix A”. Data are provided by the UN COMTRADE (2021), which is the largest depository of international trade data. It contains over 40 billion data records since 1962 and is available publicly on the internet.

The second is used to analyse the relationship that such a trade network has with COVID-19 diffusion. Data on COVID-19 diffusion come from the The European Centre for Disease Prevention and Control (2021) (ECDC), an EU agency for the protection of European citizens against infectious diseases and pandemics. The data on the distribution of COVID-19 worldwide are updated daily by the ECDC’s Epidemic Intelligence team, based on reports provided by national health authorities. Since we are interested in the first wave of the pandemic, we retrieve cross-country daily data on the number of COVID-19 infections and deaths, which we pool into five weeks from March 11st, 2020 to April 21st, 2020.

To control for other factors that can potentially affect the diffusion patterns of COVID-19, we also consider the following country-level information provided by The World Bank (2021): the real GDP per capita (GDPPC, in 2010 USD), used as a proxy for the average standard of living in a country; the total resident population (POP), taken as a proxy for a country’s size; the share of population aged 65 or higher (POP 65+); the number of hospital beds per capita available in public, private, general, and specialized hospitals and rehabilitation centres (HBEDS), which we include to capture the average quality of the health system in each country; and the average temperature in February and March (TEMP) in degrees Celsius, $^{\circ }$ C.

### Network construction

Trades between countries are represented as a weighted network, where each country is a node and connections, i.e. links between nodes, are measured by the amount of traded volume (expressed in US dollars).

At first, we separately compute the aggregate trade values of imports and exports between each pair of countries. We then consider a pair of countries (*i*, *j*) such that both imports and exports exist. Specifically, focusing on trade flows from *i* to *j*, let $w^{imp}_{ij}$ and $w^{exp}_{ij}$ be the aggregate import trade value and the aggregate export value, respectively, from *i* to *j*. We then place a weighted link from *i* to *j* representing the average value between imports and exports, defined as follows:

$$\begin{aligned} {\bar{w}}_{ij}= {\left\{ \begin{array}{ll} \frac{w^{imp}_{ij}+w^{exp}_{ij}}{2} &{}\quad \text{if}\quad w^{imp}_{ij}>0 \hspace{2mm} \text {and} \hspace{2mm} w^{exp}_{ij}>0 \\ 0 &{} \quad\text{otherwise} \end{array}\right. } \end{aligned}$$

Notice that, due to the incompleteness of the data,^5^ in general, ${\bar{w}}_{ij} \ne {\bar{w}}_{ji}$. The resulting network is then weighted and oriented, with bilateral links between two nodes eventually forming.

Since the approach of Bartesaghi et al. (2020), based on the communicability distance, has been developed on indirect networks, we investigate whether it is possible to neglect the direction of the links. To this end, we compute the Spearman correlation between in and out strength distribution for each year of the sample. The resulting correlations are 0.99 for both years. We then can substitute the bilateral arcs between nodes *i* and *j* with a single non-oriented link having weight given by the maximum value between ${\bar{w}}_{ij}$ and ${\bar{w}}_{ji}$, i.e.

$$\begin{aligned} w_{ij} = \max ({\bar{w}}_{ij},{\bar{w}}_{ji}). \end{aligned}$$

This choice is based on an information quality reason: we expected that the higher the value traded, the higher is the quality of the information that can be contained.

In Table 1, we report the global network indicators of the WTN for 2019 and 2020. As expected, the network shows an extremely connected, dense and almost complete structure. This is certainly confirmed by the average degree (51 and 52) and density^6^ (0.937 and 0.948 for 2019 and 2020, respectively). A high value of the transitivity (0.953 and 0.959 for 2019 and 2020, respectively)^7^ denotes a strong interconnection among countries. The WTN network is depicted in Figs. 8 and 9 of “Appendix B”.

**Table 1:** Global network indicators of the WTN for 2019 and 2020

Feature	2019	2020
Number of nodes	55	55
Number of links	1392	1409
Average degree	51	52
Density	0.937	0.948
Transitivity	0.953	0.959

Tables 2 and 3 show the main summary statistics and the pairwise correlations among the WTN 2019 network centrality measures and the socio-economic data, which will be used as regressors in Eq. (8). As discussed in “Econometric model” section, the WTN centrality measures and the socio-economic factors refer to 2019 to avoid the possibility that these factors might be affected by the diffusion of COVID-19.

**Table 2:** Summary statistics referred to 2019

Variable	Mean	Std. dev.	Min	Max
*Network centrality*				
Degree	50.62	5.223	27	54
Betweenness	0.0011	0.0008	0	0.0019
Local clustering	0.0022	0.0026	0.0001	0.0122
Weighted Eigenvector	0.109	0.198	0.0002	1
Strength (:10$^9$)	53.71	96.00	0.130	485.9
*Additional regressors*				
GDPPC	27085.33	26413.5	809.36	111062.3
POP (mln)	53.754	187.62	0.3613	1366.4
POP65+	0.147	0.063	0.026	0.280
HBEDS	4.031	2.375	0.600	13.40
TEMP (*C*)	6.181	10.89	− 20.99	26.80

**Table 3:** Pearson correlation matrix among regressors referred to 2019

	Degree	Betweenness	Clust. coeff.	W Eigen.	Strength	GDPPC	POP	POP65+	HBEDS	TEMP
1. Degree	1									
2. Betweenness	0.76***	1								
3. Clust. coeff.	0.45***	0.62***	1							
4. W Eigen.	0.33***	0.50***	0.87***	1						
5. Strength	0.34***	0.50***	0.96***	0.95***	1					
6. GDPPC	0.48***	0.59***	0.46***	0.42***	0.40***	1				
7. POP	0.15***	0.23***	0.23***	0.23***	0.21***	− 0.11	1			
8. POP65+	0.61***	0.51***	0.44***	0.32***	0.35***	0.51***	− 0.19***	1		
9. HBEDS	0.36***	0.16***	0.25***	0.15***	0.20***	0.20***	0.20***	0.66***	1	
10. TEMP	− 0.44***	− 0.17***	− 0.22***	− 0.29***	− 0.24***	− 0.36***	0.19***	− 0.59***	− 0.54***	1

***Significant at 1% level

## Results

In this section, we present the results emerging from the analysis of the topology of the WTN and from the econometric regressions. The former makes a resilient mesoscale structure emerge in the international trade among countries, while in the latter, we show that both countries’ and communities’ centrality are good predictors of the risk of COVID-19 infections and deaths. In line with our expectations, this can explain the reason why one country was more vulnerable than another during the first wave of the pandemic.

### Evolution of WTN during COVID-19

We apply the methodology described in “Community detection based on communicability distance” section by using the communicability distance. As already stressed in the previous sections, the WTN is characterized by an almost complete structure, where direct connections between countries are dominant. With this approach, we have a tool to quantify the depth of the level of communication between countries. At first, we compute the communicability distance between countries for networks in 2019 and 2020 by applying Formula (4). In 2019, the nearest countries are Canada and the United States ($\xi _{\min }= 1.1166$), and they still remain in 2020 ($\xi _{\min }= 1.1316$). In 2019, the farthest countries were Kyrgyzstan and the United States ($\xi _{\max }=1.4969$), whereas in 2020, the farthest were the United States and Iceland ($\xi _{\max }=1.5077$). These results underline the central role of the United States in the WTN and at the beginning of the pandemic.

**Fig. 1:**
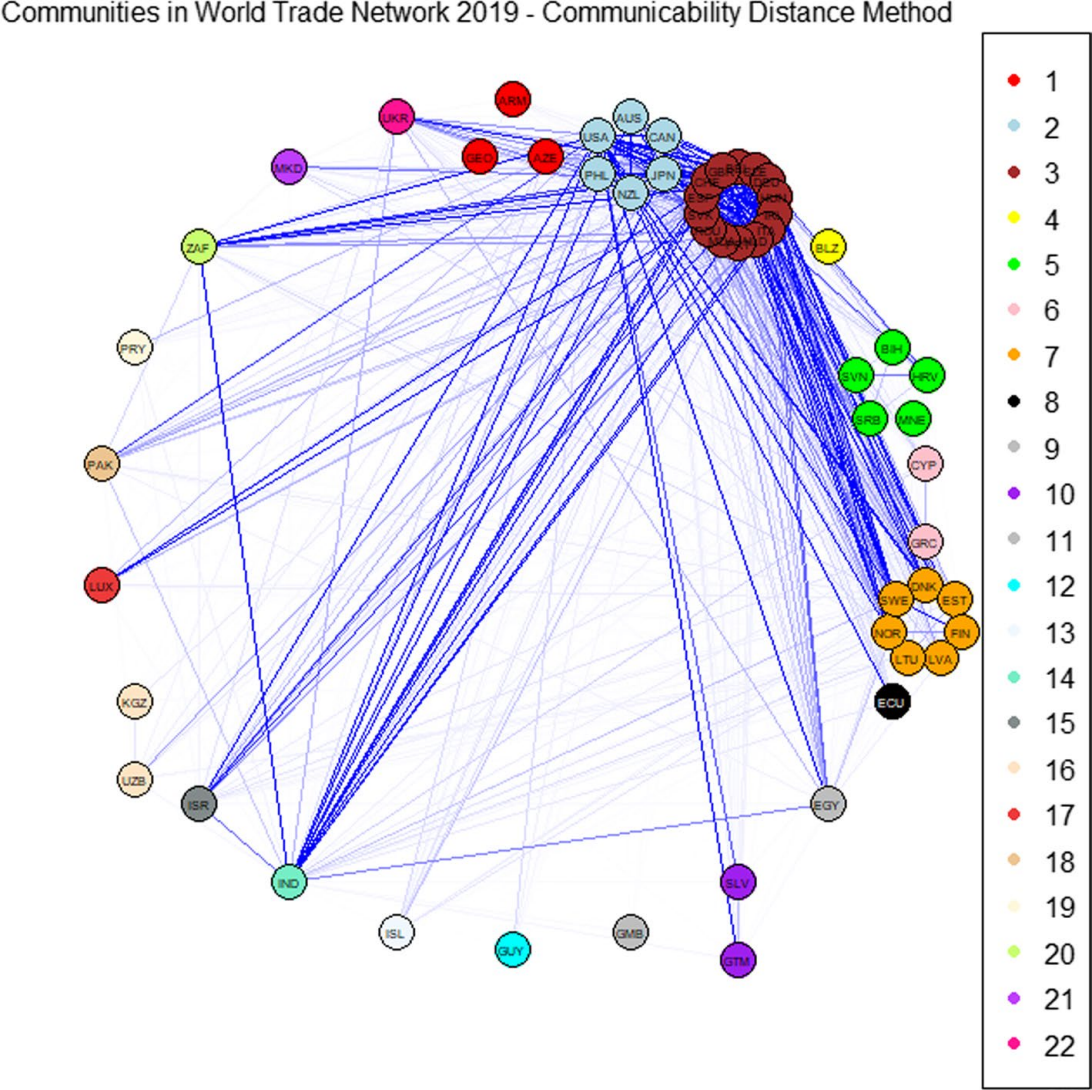
Community graph obtained with the communicability distance method in 2019

**Fig. 2:**
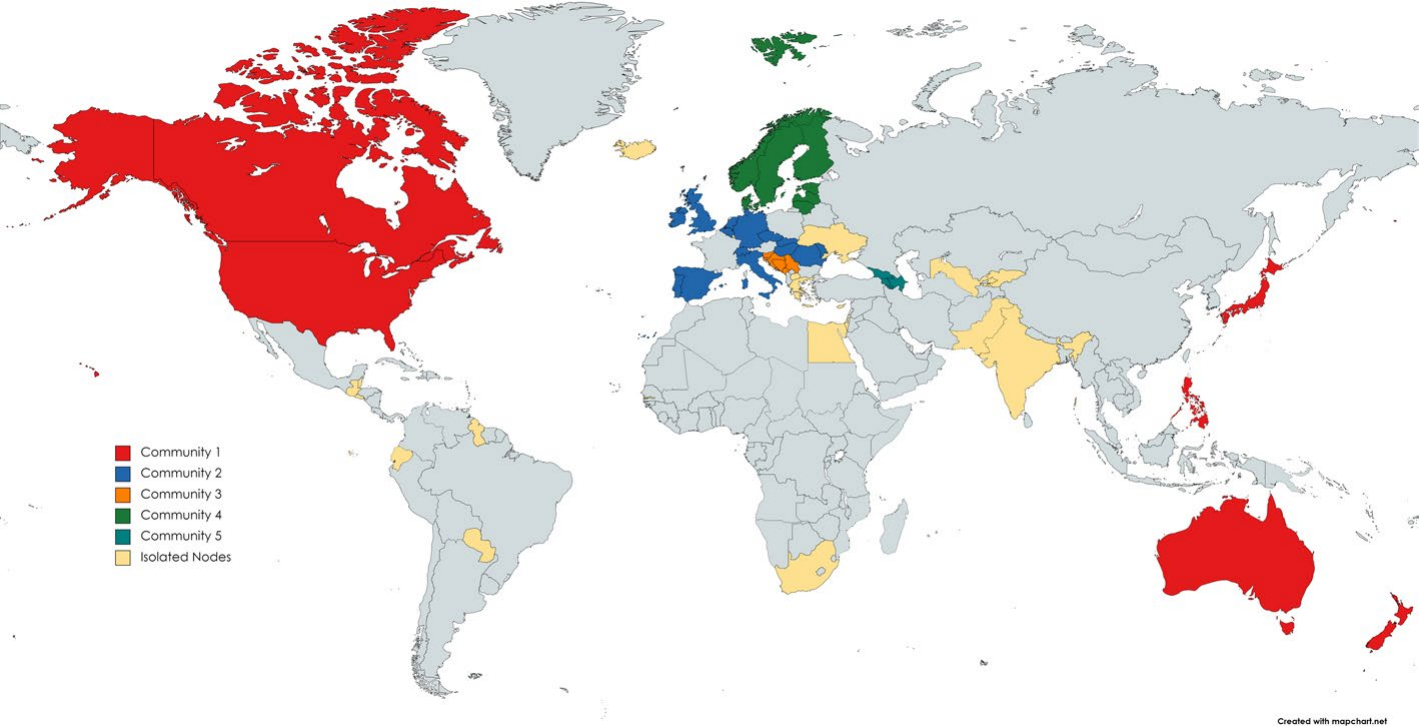
World map with the optimal community structure in 2019

**Fig. 3:**
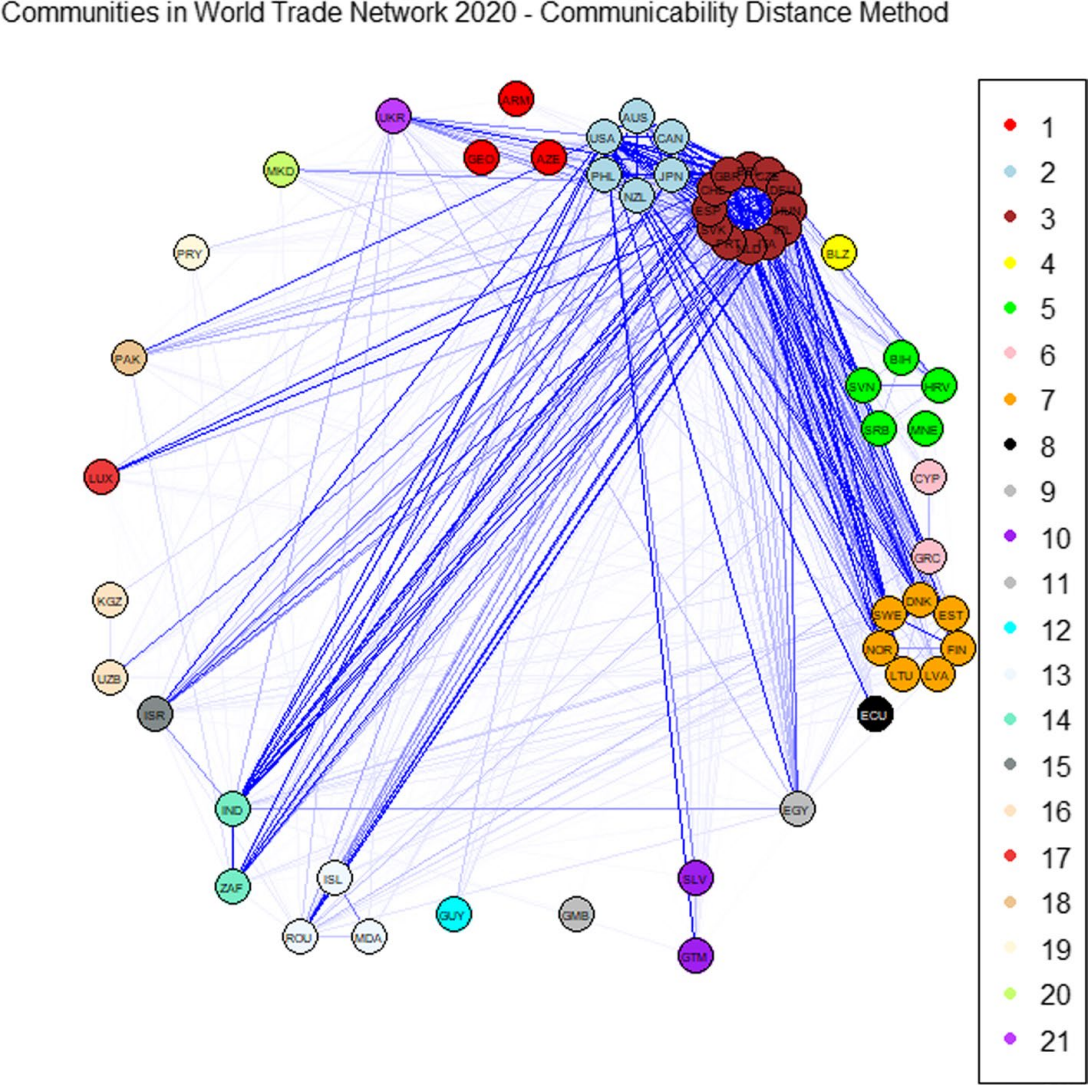
Community graph obtained with the communicability distance method in 2020

**Fig. 4:**
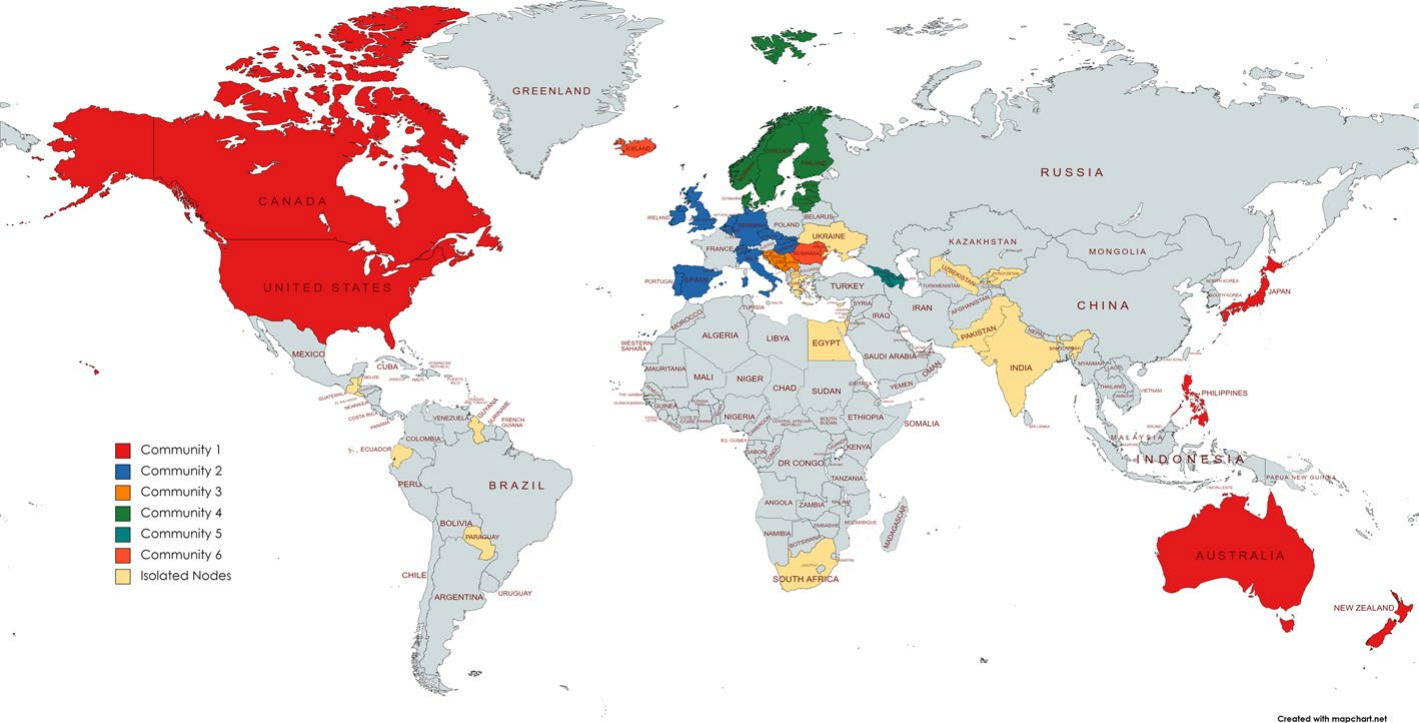
World map with the optimal community structure in 2020

We then apply the community detection method based on the communicability distance. The optimal thresholds $\xi _0^*$ maximising the quality function *Q* defined in Eq. (6) are 1.3676 and 1.3723 for 2019 and 2020, respectively. The corresponding optimal values of the quality function $Q^*$ are 86.2301 and 87.0466 for 2019 and 2020, respectively. Figures 1 and 3 report the community graphs whose adjacency matrices are computed according to Formula (5). Communities obtained with the optimal threshold $\xi _0^*$ for both years are also shown in Figs. 2 and 4, through a world map representation.

Grey countries are those not included in the sample, while the yellow countries are the isolated nodes, which, in the following, will be denoted as the rest of the world.

Observe that, in 2019, the number of communities is 22 with 18 isolated nodes, whereas in 2020, this number is 21 with 16 isolated nodes. The slight reduction in the number of communities can be explained by the effect of a strengthening of long-range alliances: on the one hand, countries reinforce the existing links, and on the other hand, they make deals with geographic neighbours.

In correspondence with the optimal threshold $\xi _0^*$, community detection in the WTN shows a fragmented structure, where three strong communities emerge. The first is the European community, containing 12 European countries; the second contains the United States, Canada, Japan and Australia; and the third community includes the North European group. The member of these communities are listed in Table 4 for 2019 and in Table 5 for 2020.

**Table 4:** Members of the five most populous communities in 2019

	Size	Members
Community 1	6	AUS CAN JPN NZL PHL USA
Community 2	14	BEL CHE CZE DEU ESP GBR HUN
		IRL ITA MDA NLD PRT ROU SVK
Community 3	5	BIH HRV MNE SRB SVN
Community 4	7	DNK EST FIN LTU LVA NOR SWE
Community 5	3	ARM AZE GEO

**Table 5:** Members of the six most populous communities in 2020

	Size	Members
Community 1	6	AUS CAN JPN NZL PHL USA
Community 2	12	BEL CHE CZE DEU ESP GBR HUN
		IRL ITA NLD PRT SVK
Community 3	5	BIH HRV MNE SRB SVN
Community 4	7	DNK EST FIN LTU LVA NOR SWE
Community 5	3	ARM AZE GEO
Community 6	3	ISL MDA ROU

Not surprisingly, we can also observe a persistence of the community structure in the WTN during the pandemic situation. The type of data (average total trade exchanged) do not allow us to show important movements. We expect that, by focusing on some specific sectors (such as, for example, the pharmaceutical industry), possible community changes could be noticed; however, at this time, the available data do not allow us to do this.

Moreover, the analysis period starts in January and ends in June. Those months in 2020 contain only the beginning of the COVID-19 pandemic, i.e. the so called ’first wave’. Reasonably, the COVID-19 pandemic situation cannot be reflected immediately in the trade volumes, and it is not possible to see the effects of the containment measures.

We now compare the results obtained with the methodology proposed in Bartesaghi et al. (2020) with a classical methodology in community detection, the Louvain method (Blondel et al. 2008). This method is based on the maximisation of a modularity score for each community, where the modularity function quantifies the quality of an assignment of nodes to communities. We observe that the classical method provides a less detailed division in the WTN. In both years, we can observe three communities: the first one contains Europe, the second one contains the United States and Pacific area and the third corresponds to Northern Europe.

**Fig. 5:**
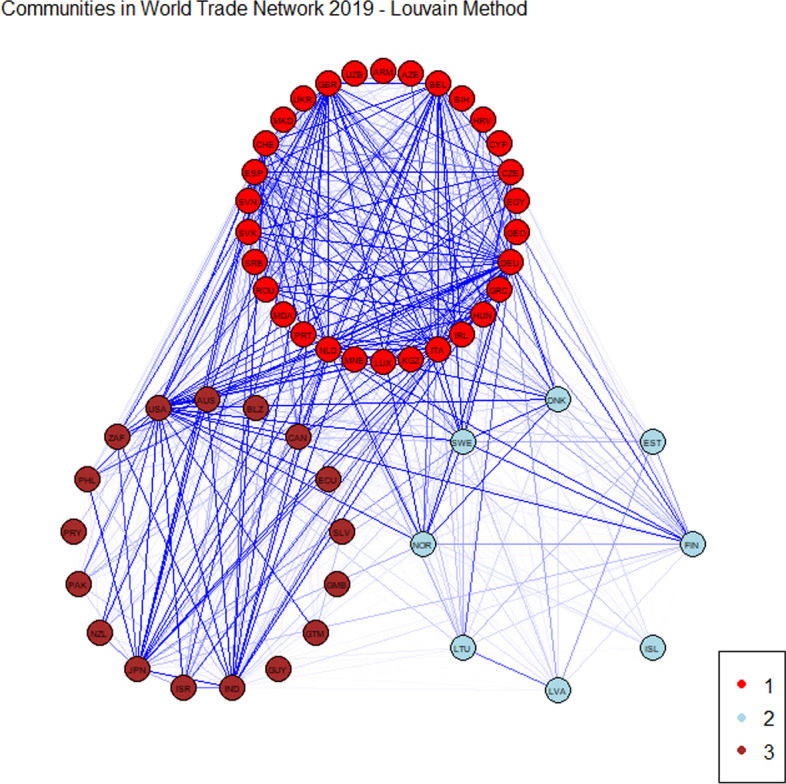
Communities obtained with the Louvain method referred to 2019

**Fig. 6:**
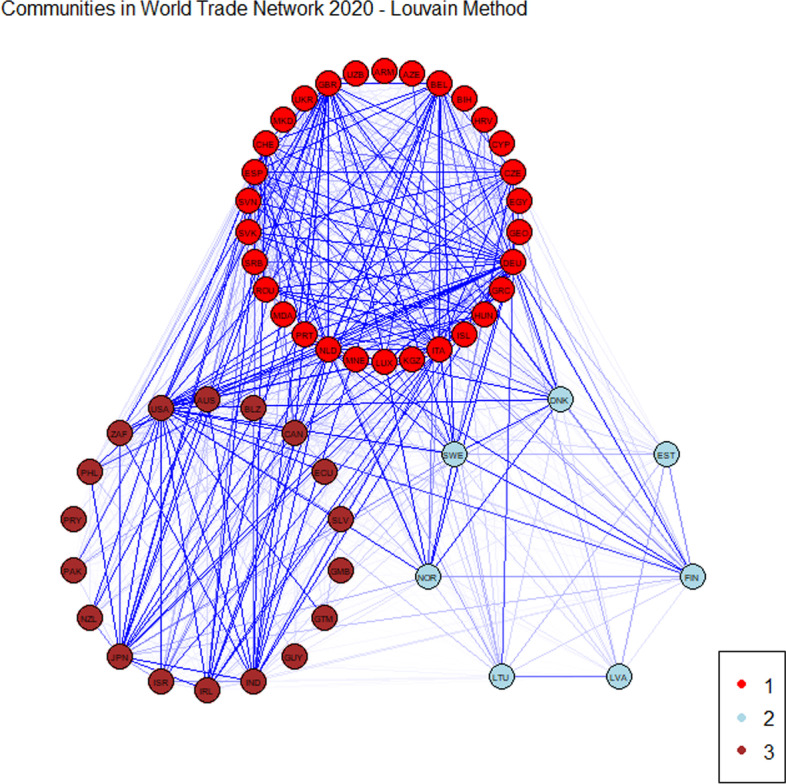
Communities obtained with the Louvain method referred to 2020

Figures 5 and 6 report the communities obtained by applying the Louvain method for the year 2019 and 2020, respectively.

“Appendix C” shows in detail the results obtained with this method. Members of communities are plotted in Figs. 10 and 11 for 2019 and 2020, respectively. The two lists of countries collected into communities are shown in Tables 12 and 13 for 2019 and 2020, respectively.

We notice that, as with the methodology based on communicability distance, the Louvain method also reveals a structural persistence in the WTN during the first wave of the COVID-19 pandemic. This is in line with the recent results obtained by Kiyota (2022). We emphasise that the classical method catches only a persistence in the macroscopic structure, while the method of Bartesaghi et al. (2020) reveals a persistence in the mesoscale structure. This result confirms that by looking beyond the direct connections, it is possible to capture a strong interactions between countries.

### Impact of countries’ centrality measures on the COVID-19 pandemic

#### Baseline econometric model

Tables 6 and 7 show the results of regressions (as in Eq. (8)) concerning, respectively, the number of COVID-19 infections and the number of COVID-19 deaths. In both tables, each column reports the results of a regression based on a model that uses the five different TNC indicators introduced in “Econometric model” section one at a time.

In Table 6, we see that the IRRs of all our centrality indicators are always statistically significant and higher than 1: in general, a higher country centrality in the international trade network corresponds to a higher risk of infection.

Specifically, we find that, ceteris paribus, a one unit increase in each TNC indicator is associated with an expected increase in the risk of infection by a factor ranging from 3.2 (Column 4 referred to the weighted eigenvector) to 4.2 (Column 3 referred to the local clustering coefficient). Moreover, we note that the IRR of each TNC indicator is always higher than the IRR of each other regressor, meaning that a country’s centrality in the WTN is a key variable when analysing the initial diffusion of COVID-19.

Interestingly, the AIC and BIC statistics, in line with the value of the pseudo log-likelihood and the pseudo $R^2$, show that the model with the highest goodness of fit with respect to the observed number of infections is that of Column 3, where the TNC indicator corresponds to the local clustering coefficient. Incidentally, the IRR of this latter is also the highest among all the other TNC indicators.

**Fig. 7:**
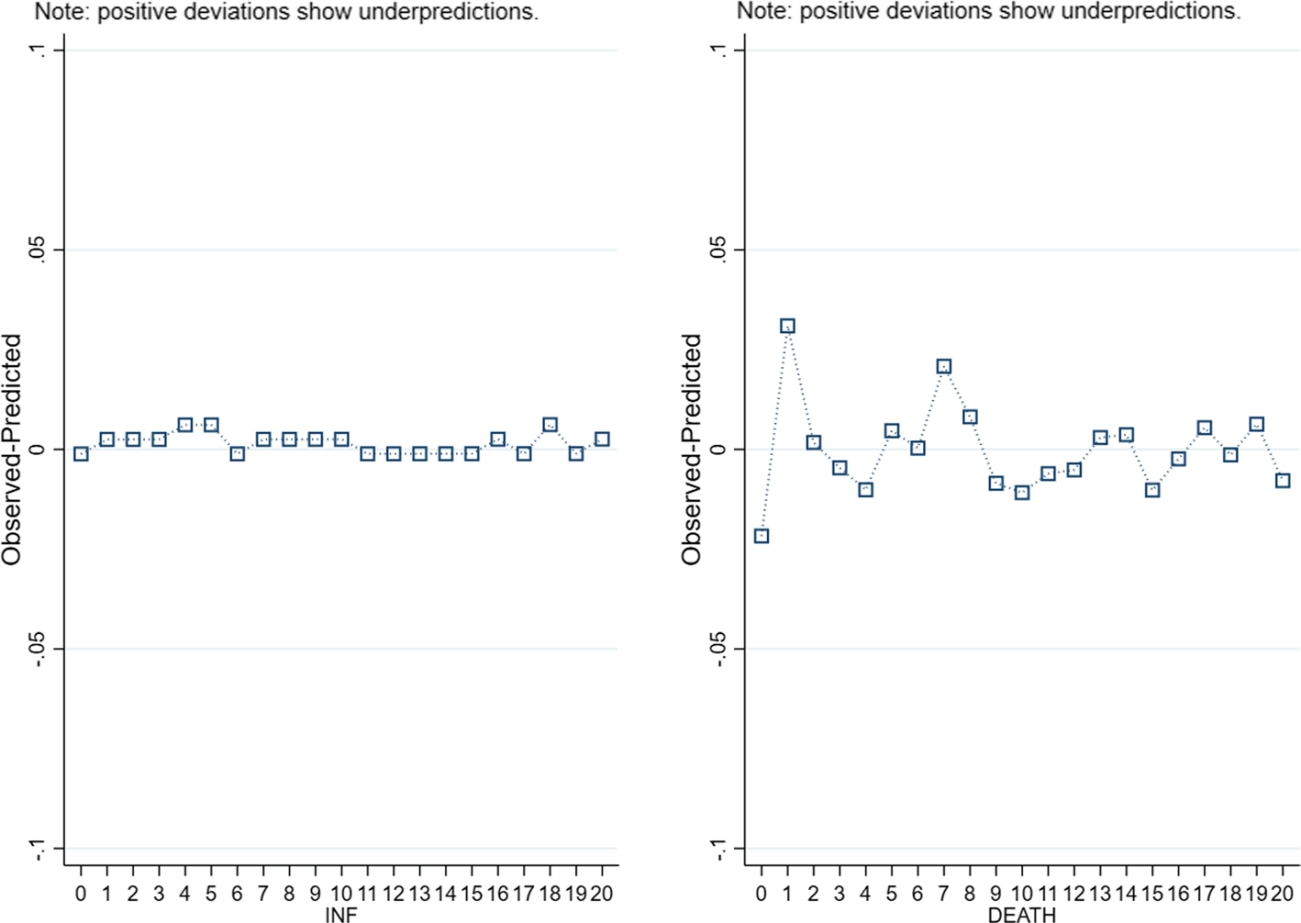
Difference between observed and predicted INF (left) and DEATH (right)

**Fig. 8:**
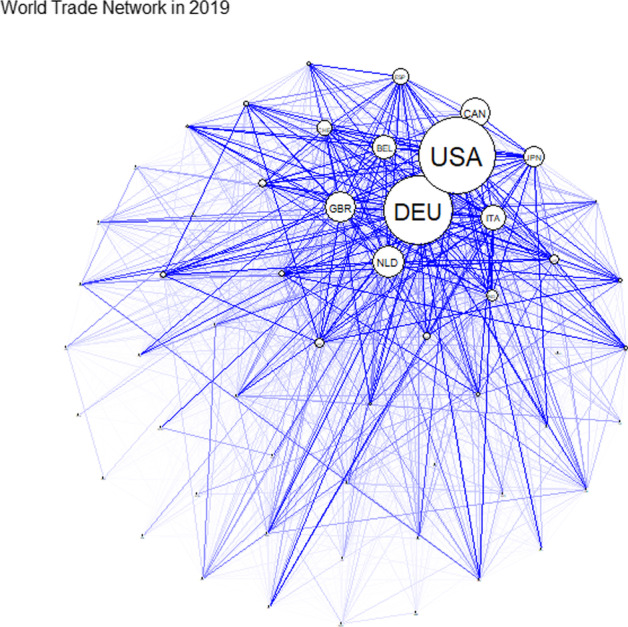
WTN representation in 2019. The size of the nodes is proportional of its strength

**Fig. 9:**
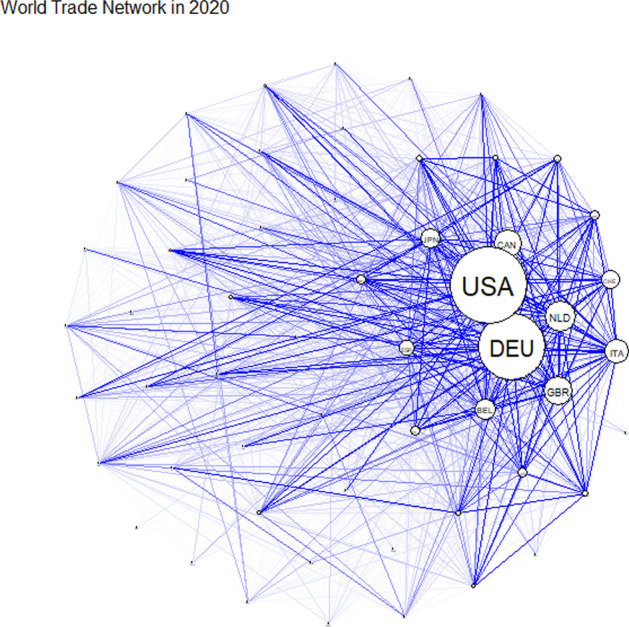
WTN representation in 2020. The size of the nodes is proportional of its strength

**Fig. 10:**
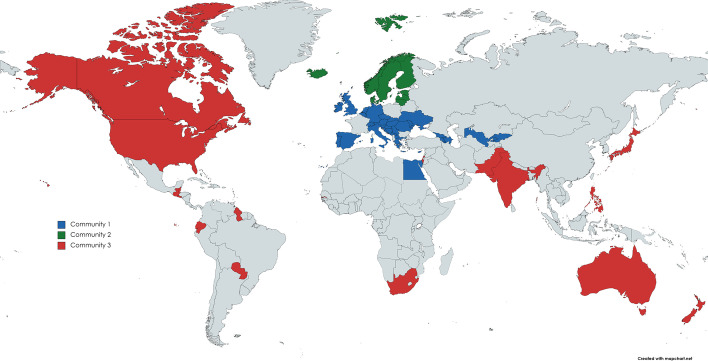
World map with the communities obtained by applying the Louvain method in 2019

**Fig. 11:**
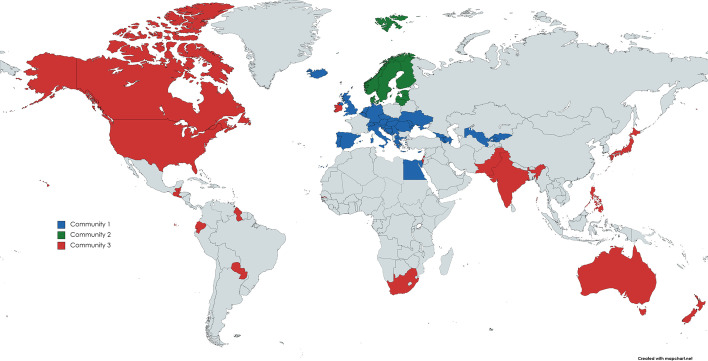
World map with the communities obtained by applying the Louvain method in 2020

In addition, both in Table 6 and in Table 7, we find that the Pearson goodness of fit test always rejects the null hypothesis that the sample mean equals the sample variance, leading to the conclusion that a negative binomial regression model fits our data better than a Poisson model does. The validity of our models is also confirmed in Fig. 7, where we plot the differences between observed and predicted infections (left chart) and deaths (right chart) for a maximum number of 20. Comparing the two charts, we find that these differences are lower for the number of infections, for which the average difference over the total number of cases is of the order of 0.002. In any case, even the difference between the observed and the predicted number of deaths remains low, e.g. of the order of 0.008 across the entire distribution.

Looking at the other regressors, we find that the risk of infection increases with the country’s GDP per capita (Columns 3–5), with the share of elderly population (Columns 1 and 3–5) and with a lower endowment of health facilities (Columns 3–5), confirming previous results obtained for a wider set of countries (Antonietti et al. 2021a).

**Table 6:** Negative binomial regressions: infections, incidence rate ratios

DEP. VAR.: INF	(1)	(2)	(3)	(4)	(5)
Degree	3.608***				
	(0.631)				
Betweenness		4.121***			
		(1.868)			
Local Clustering			4.201***		
			(0.968)		
Weighted Eigenvector				3.225***	
				(0.875)	
Strength (:10^9^)					3.339***
					(1.052)
GDPPC	1.676	1.147	1.191**	1.238*	1.264**
	(0.632)	(0.266)	(0.103)	(0.144)	(0.133)
POP	3.026	1.813	1.023	1.132	1.136
	(3.227)	(2.130)	(0.134)	(0.224)	(0.184)
POP65+	2.226***	1.685	1.688***	2.680***	2.420***
	(0.685)	(0.539)	(0.294)	(0.636)	(0.564)
HBEDS	0.678	0.710	0.585***	0.557**	0.555***
	(0.212)	(0.230)	(0.087)	(0.132)	(0.107)
TEMP	1.120	0.728	1.080	1.387	1.316
	(0.226)	(0.335)	(0.142)	(0.350)	(0.231)
Week dummies	Yes	Yes	Yes	Yes	Yes
Overdispersion $(\alpha )$	1.319***	1.393***	0.998***	1.263***	1.193***
	(0.209)	(0.170)	(0.178)	(0.213)	(0.204)
N	275	275	275	275	275
Log Pseudo-likelihood	− 2490.6	− 2500.5	− 2440.5	− 2482.5	− 2472.2
AIC	5005.1	5025.1	4905.1	4989.0	4968.4
BIC	5048.5	5068.5	4948.4	5032.4	5011.82
Pseudo $R^2$	0.076	0.072	0.094	0.079	0.083
Max VIF	3.01	3.20	2.72	2.57	2.60
Mean VIF	1.79	1.89	1.73	1.69	1.68
Pearson GOF test	1.34e+07	1.29e+07	2061931	2680124	2229799
*p*-value	0.000	0.000	0.000	0.000	0.000

Country-level clustered standard errors in parentheses.

All the estimates also include a constant term. *$p < 0.1$; **$p < 0.05$; ***$p < 0.01$

The same kinds of results emerge for the case of COVID-19 fatalities, as shown in Table 7. Again, we find that the higher a country’s centrality in the trade network, the higher the risk of death due to COVID-19. Ceteris paribus, if a country’s centrality increases by one unit, the risk of death is expected to increase by a factor ranging from 2.9 (Column 1) to 8.6 (Column 3). Again, the AIC and BIC statistics show that the econometric model that uses the local clustering coefficient is the one with the highest goodness of fit with respect to the observed number of deaths.

In addition, and still in line with previous literature (Antonietti et al. 2021a), we find that the risk of death increases with the share of elderly population and with a lower endowment of hospital beds in a country. On top of this, both in Table 6 and in Table 7, the VIF statistics are low enough with respect to the commonly accepted threshold of 5, showing again that multicollinearity is not an issue.

**Table 7:** Negative binomial regressions: deaths, incidence rate ratios

DEP. VAR.: DEATH	(1)	(2)	(3)	(4)	(5)
Degree	2.878***				
	(0.888)				
Betweenness		6.750***			
		(3.022)			
Local Clustering			8.648***		
			(3.579)		
Weighted Eigenvector				5.123**	
				(3.339)	
Strength (:$10^9$)					6.893***
					(5.123)
GDPPC	1.458	0.755	0.930	0.933	0.966
	(0.671)	(0.162)	(0.141)	(0.149)	(0.136)
POP	2.897	1.486	0.867	1.020	1.016
	(3.258)	(1.636)	(0.102)	(0.196)	(0.169)
POP65+	6.223***	3.099***	2.136***	5.423***	4.037***
	(2.549)	(1.216)	(0.606)	(2.338)	(1.830)
HBEDS	0.370***	0.434***	0.484***	0.382***	0.429***
	(0.105)	(0.127)	(0.099)	(0.119)	(0.127)
TEMP	1.721	0.973	1.426*	1.968	1.915**
	(0.471)	(0.424)	(0.272)	(0.814)	(0.556)
Week dummies	Yes	Yes	Yes	Yes	Yes
Overdispersion ($\alpha$)	2.245***	2.055***	1.400***	1.933***	1.805***
	(0.283)	(0.255)	(0.217)	(0.266)	(0.263)
N	275	275	275	275	275
Log Pseudo-likelihood	− 1519.4	− 1500.6	− 1437.9	− 1490.9	− 1479.2
AIC	3062.9	3025.3	2899.9	3005.7	2982.3
BIC	3106.3	3068.7	2943.3	3049.2	3025.7
Pseudo $R^2$	0.101	0.112	0.149	0.118	0.124
Max VIF	3.01	3.20	2.72	2.57	2.60
Mean VIF	1.79	1.89	1.73	1.69	1.68
Pearson GOF test	1.07e+07	713491.1	228495.4	297189.9	301596.9
*p*-value	0.000	0.000	0.000	0.000	0.000

Country-level clustered standard errors in parentheses.

All the estimates also include a constant term. *$p < 0.1$; **$p < 0.05$; ***$p < 0.01$.

#### Econometric result considering WTN mesoscale structure

The results of the econometric analysis with the community average clustering coefficient are shown in Table 8. Columns 1 and 3 report the results shown in Column 3 of Tables 6 and 7 concerning infections (INF) and deaths (DEATH), respectively, while Columns 2 and 4 show the new results for the model that uses average community coefficient ${\bar{C}}_k$ as the main regressor.

Interestingly, we find that a unit increase in averaged local clustering coefficient ${\bar{C}}_k$ corresponds to a higher risk of infection and death as compared to country *i* local clustering coefficient $C_i$. Those risks pass from an order of 4.2 to 5.6 in the case of infection and from an order of 8.6 to 10.3 in the case of death. These results confirm that community-specific measures of country centrality can provide even stronger results on the first-wave diffusion patterns of COVID-19. The fact that communities are detected using a wider set of trade-related information between countries, which implicitly include unobserved flows of people other than merchandise, allows us to account for a higher risk of contagion attributable to international trade.

Finally, Tables 9 and 10 show the results of our additional robustness tests. As specified in “Econometric model considering WTN mesoscale structure" section, we have re-estimated Eq. 8 for each single week from March 11th to April 21st 2020, using five negative binomial regression models on a corresponding sample of 55 countries. To save space, each column reports only the IRR of the Local Clustering variable among the TNC indicators. We have also estimated each of the five equations using the Average Community Clustering as the main regressor, and in both tables, we only report the corresponding IRR in order to compare it with that of the Local Clustering. Three interesting results emerge from both of the tables. First, the estimates confirm that the IRR of Local Clustering is highly statistically significant, larger than 1 and of an order of magnitude comparable to that obtained in Tables 6 and 7, Column 3. Second, we observe that the IRR of Local Clustering increases over the weeks in Table 9 and decreases in Table 10, which means that a country’s centrality in the WTN correlates with the risk of infection more and more intensively as the pandemic spreads over time, whereas the correlation with the death risk becomes less and less intense. Third, we also find that the IRR of the Average Community Clustering is of the same magnitude as that obtained in Table 8 and always larger than the IRR of Local Clustering.

**Table 8:** General versus average community clustering coefficient and COVID-19 diffusion

DEP. VAR.:	INF		DEATH	
	(1)	(2)	(3)	(4)
Local clustering	4.201***		8.648***	
	(0.968)		(3.579)	
Average community clustering		5.551***		10.25***
		(2.486)		(4.977)b
GDPPC	1.191**	1.510**	0.930	1.166
	(0.103)	(0.243)	(0.141)	(0.199)
POP	1.023	2.499	0.867	2.107
	(0.134)	(2.467)	(0.102)	(2.137)
POP65+	1.688***	1.705**	2.136***	2.945***
	(0.294)	(0.425)	(0.606)	(0.904)
HBEDS	0.585***	0.652*	0.484***	0.419***
	(0.087)	(0.146)	(0.099)	(0.087)
TEMP	1.080	0.980	1.426*	1.294
	(0.142)	(0.286)	(0.272)	(0.448)
Week dummies	Yes	Yes	Yes	Yes
Overdispersion ($\alpha$)	0.998***	1.344***	1.400***	1.898***
	(0.178)	(0.211)	(0.217)	(0.284)
N	275	275	275	275
Pseudo $R^2$	0.094	0.074	0.149	0.120

Country-level clustered standard errors in parentheses.

All the estimates also include a constant term.

*$$p < 0.1$$; **$$p < 0.05$$; ***$$p < 0.01$$

**Table 9:** Robustness tests on infections (INF) by weekly estimates

DEP. VAR.: INF	Week 1	Week 2	Week 3	Week 4	Week 5
Local Clustering	3.985***	4.257***	4.180***	4.307***	4.327***
	(0.880)	(0.969)	(0.927)	(1.003)	(1.042)
Average Community Clustering	5.160***	6.066***	5.713***	5.613***	5.362***
	(2.560)	(2.824)	(2.521)	(2.417)	(2.318)
GDPPC	1.405***	1.302***	1.174*	1.087	1.011
	(0.120)	(0.115)	(0.108)	(0.097)	(0.094)
POP	0.950	0.911	1.006	1.053	1.110
	(0.162)	(0.112)	(0.131)	(0.128)	(0.150)
POP65+	2.148***	1.878***	1.707***	1.517**	1.414**
	(0.360)	(0.311)	(0.304)	(0.285)	(0.287)
HBEDS	0.470***	0.528***	0.566***	0.631***	0.674**
	(0.060)	(0.077)	(0.086)	(0.097)	(0.111)
TEMP	1.208	1.173	1.056	1.029	0.979
	(0.203)	(0.146)	(0.136)	(0.144)	(0.142)
Overdispersion ($\alpha$)	0.948***	0.957***	0.963***	0.955***	1.002***
	(0.191)	(0.191)	(0.186)	(0.174)	(0.176)
N	55	55	55	55	55
Pseudo $R^2$	0.108	0.096	0.089	0.084	0.079

Country-level clustered standard errors in parentheses.

All the estimates also include a constant term.

*$$p < 0.1$$; **$$p < 0.05$$; ***$$p < 0.01$$.

**Table 10:** Robustness tests on deaths (DEATH) by weekly estimates

DEP. VAR.: DEATH	Week 1	Week 2	Week 3	Week 4	Week 5
Local Clustering	11.23***	8.890***	7.681***	7.837***	7.974***
	(5.953)	(3.903)	(2.887)	(2.854)	(2.898)
Average Community Clustering	10.09***	10.53***	9.609***	9.985***	9.838***
	(5.996)	(5.640)	(4.460)	(4.670)	(4.550)
GDPPC	1.147	1.025	0.900	0.898	0.852
	(0.219)	(0.163)	(0.129)	(0.121)	(0.108)
POP	0.805	0.805*	0.846	0.910	0.940
	(0.157)	(0.103)	(0.089)	(0.096)	(0.104)
POP65+	2.232**	2.481***	2.251***	1.979**	1.788**
	(0.908)	(0.726)	(0.595)	(0.530)	(0.497)
HBEDS	0.475***	0.440***	0.460***	0.504***	0.514***
	(0.103)	(0.092)	(0.099)	(0.116)	(0.113)
TEMP	2.130***	1.719***	1.332	1.272	1.112
	(0.488)	(0.359)	(0.257)	(0.252)	(0.228)
Overdispersion ($\alpha$)	1.851***	1.430***	1.232***	1.241***	1.260***
	(0.343)	(0.287)	(0.206)	(0.188)	(0.181)
N	55	55	55	55	55
Pseudo $R^2$	0.177	0.156	0.143	0.130	0.122

Country-level clustered standard errors in parentheses.

All the estimates also include a constant term.

*$$p < 0.1$$; **$$p < 0.05$$; ***$$p < 0.01$$.

## Conclusions

In this paper, we evaluate the relationship between the WTN’s structure and the first wave of COVID-19. The complex nature of the trade relationships between countries requires them to be investigated using effective network tools to reveal the hidden mesoscale structure, which is characterised by strong interconnections, as well as to assess countries’ central positions in the network. These trade relationships could have been impacted by the COVID-19 pandemic, both directly because of the spread of the virus and indirectly due to the policies that countries have implemented to reduce the pandemic’s diffusion and consequences. At the same time, it is very possible that the pandemic itself has been favoured by the complex network of relationships are established when trade occurs. Through network measures, we have evaluated the extent to which the WTN has been affected by COVID-19 and the extent to which countries’ centrality explains the diffusion and mortality of COVID-19. Moreover, we have shown that the WTN’s mesoscale structure has been resilient to the diffusion of the pandemic. Even if such a result was expected at the global level, we have shown that it holds also when looking at the number and members of the communities that emerged before and during the outbreak of COVID-19, showing that the strength of long-range alliances have not been affected by the beginning of the COVID-19 pandemic.

On the contrary, country centrality has shown to be a key explanatory variable for the diffusion and mortality of COVID-19. We showed that country centrality measures strongly explained the risk of infection and mortality, when controlling for other possible confounding socio-economic factors.

Both results can be of interest for the analysis of the structure and evolution of the WTN and from the point of view of studying the determinants and the consequences of the COVID-19 pandemic. International trade activities are also related to human factors that have been crucial in the spread of the pandemic. In future research, it could be interesting to deeply investigate this aspect, comparing the different roles of human-based and economic linkages in COVID-19 diffusion. Finally, the established link between the pandemic and the structure of the network of international trade can provide useful policy insights. More precisely, this knowledge can guide decision makers about the adoption and calibration of relevant public safety policies, such as general lockdown measures and temporary trade bans, which have huge economic consequences but have shown so far unclear effectiveness on the virus’ spread. This can be important for both the actual COVID-19 pandemic, which at the time of the writing of this article is still widely diffused worldwide, as well as for the unfortunate yet possible case of the diffusion of a new pandemic event in the future.

## Data Availability

The datasets used and/or analysed during the current study are available from the corresponding author on reasonable request.

## Appendix A: List of countries

See Table 11.

**Table 11:** List of countries of the WTN

Country	Code
Armenia	ARM
Australia	AUS
Azerbaijan	AZE
Belgium	BEL
Belize	BLZ
Bosnia Herzegovina	BIH
Canada	CAN
Croatia	HRV
Cyprus	CYP
Czech Rep.	CZE
Denmark	DNK
Ecuador	ECU
Egypt	EGY
El Salvador	SLV
Estonia	EST
Finland	FIN
Gambia	GMB
Georgia	GEO
Germany	DEU
Greece	GRC
Guatemala	GTM
Guyana	GUY
Hungary	HUN
Iceland	ISL
India	IND
Ireland	IRL
Israel	ISR
Italy	ITA
Japan	JPN
Kyrgyzstan	KGZ
Latvia	LVA
Lithuania	LTU
Luxembourg	LUX
Montenegro	MNE
Netherlands	NLD
New Zealand	NZL
Norway	NOR
Pakistan	PAK
Paraguay	PRY
Philippines	PHL
Portugal	PRT
Rep. Of Moldova	MDA
Romania	ROU
Serbia	SRB
Slovakia	SVK
Slovenia	SVN
South Africa	ZAF
Spain	ESP
Sweden	SWE
Switzerland	CHE
TFYR of Macedonia	MKD
Ukraine	UKR
United Kingdom	GBR
United States	USA
Uzbekistan	UZB

## Appendix B: World trade network representation

This section collects the WTN representations in 2019 and 2020.

See Figs. 8 and 9.

## Appendix C: Community detection based on Louvain method

The world map of communities obtained by applying the Louvain method for 2019 and 2020 are plotted in Figs. 10 and 11, respectively. Tables 12 and 13 list the members of communities.

**Table 12:** Members of the communities detected by applying the Louvain method in 2019

	Size	Members
Community 1	30	ARM AZE BEL BIH CHE CYP CZE DEU EGY ESP
		GBR GEO GRC HRV HUN IRL ITA KGZ LUX MDA
		MKD MNE NLD PRT ROU SRB SVK SVN UKR UZB
Community 2	7	DNK EST ISL LTU LVA NOR SWE
Community 3	18	AUS BLZ CAN ECU GMB GTM GUY IND ISR JPN
		NZL PAK PHL PRY SLV USA ZAF

**Table 13:** Members of the communities detected by applying the Louvain method in 2020

	Size	Members
Community 1	30	ARM AZE BEL BIH CHE CYP CZE DEU EGY ESP
		GBR GEO GRC HRV HUN ISL ITA KGZ LUX MDA
		MKD MNE NLD PRT ROU SRB SVK SVN UKR UZB
Community 2	7	DNK EST FIN LTU LVA NOR SWE
Community 3	18	AUS BLZ CAN ECU GMB GTM GUY IND IRL ISR
		JPN NZL PAK PHL PRY SLV USA ZAF

## Abbreviations

WTN: World Trade Network
VIF: Variance Inflation Factor
INF: Number of infections
DEATH: Number of deaths
GDP: Gross domestic product
GDPPC: Gross domestic product per capita
POP: total resident population
POP65+: Share of elderly population
HBEDS: Number of hospital beds
AIC: Akaike Information Criterion
BIC: Bayesian Information Criterion
IRR: Incidence rate ratio
TNC: Trade network centrality

## Footnotes

1 As it will be seen in Table 3, network centrality measures are highly correlated and, therefore, we use network centrality measures one at a time in Eq. (8).

2 We have also estimated Eq. (8) without the week dummies, and we do not find any relevant change in the results. Rather, we find that the goodness of fit of the model decreases. The results are not available here for reasons of space but are available upon request.

3 The VIF is the ratio of variance in a model that uses multiple independent variables and the variance of a model that uses only one independent variable. This statistic is used to test for the severity of multicollinearity in linear regressions. To see in detail how VIF statistic works, we refer, for instance, to James et al. (2013).

4 We notice that the list of countries does not contain France, Russia and China, which are among the largest in terms of trade. The reason lies in the lack of WTN data from UN COMTRADE (2021) for these countries for the first half of 2020. However, concerning these countries, we expect that community detection results are in line with those obtained for the year 2016 by Bartesaghi et al. (2020), as the network nature is the same, as well as the applied methodology. The authors show that France belongs to the European cluster. China is one of the major players in the Pacific cluster, while Russia belongs to the Baltic cluster.

5 Since we refer to 2019 and 2020, not all countries have completely communicated their data to UN Comtrade.

6 Density measures how many links between nodes exist compared to how many links between nodes are possible.

7 The transitivity coefficient is the ratio between the number of actual triangles and the number of potential ones (see Newman (2001)). It expresses the network cliquishness, as it can be seen as the probability that the adjacent nodes of a reference node are themselves connected.
